# Incidence of Acute Pulmonary Edema Before and After the Systematic Use of Ultrasound B-Lines

**DOI:** 10.3390/jpm14111094

**Published:** 2024-11-06

**Authors:** Alessandra Urso, Rocco Tripepi, Sabrina Mezzatesta, Maria Carmela Versace, Giovanni Luigi Tripepi, Vincenzo Antonio Panuccio

**Affiliations:** 1 Nephrology, Dialysis and Transplantation Unit, Grande Ospedale Metropolitano “Bianchi-Melacrino-Morelli”, 89124 Reggio Calabria, Italy; alessandra.urso@ospedalerc.it; 2 Institute of Clinical Physiology, National Research Council CNR-IFC, 89124 Reggio Calabria, Italysabrinamezzatesta@cnr.it (S.M.);

**Keywords:** acute pulmonary edema, dialysis, US B-lines

## Abstract

Introduction: Acute pulmonary edema (APE) due to fluid overload is considered the most feared complication in hemodialysis patients. Various diagnostic tests have been proposed to assess the fluid status in patients with end-stage kidney failure (ESKF); among these, lung ultrasound (measuring the number of B-lines) is emerging as a promising tool to identify pulmonary congestion in this patient population. Methods: We compared the incidence of APE before and after the implementation of lung ultrasound as a routine practice in our unit. The pre (from 1 January 2007 to 31 December 2008)- and post (from 1 January 2017 to 31 December 2018)-B-line implementation periods included 98 and 108 hemodialysis patients, respectively. By accurately reviewing their electronic medical records, all episodes of APE were collected. The 10-year interval between the two periods was specifically chosen to ensure no overlap between patients of the two cohorts whereas the single-center design was adopted to minimize the influence of center effect on the study results. Results: APE episodes occurred more frequently in patients from the pre-B-line implementation group (18/98, i.e., 18.4%) compared with those from the post B-line implementation group (6/108, i.e., 5.5%) (*p* = 0.004). An analysis based on repeated APE events showed that the incidence rate of APE was significantly higher during the pre-implementation period (2.0 APE episodes per 100 person-months, 95% CI: 1.4–2.7) than during the post-implementation period (0.3 APE episodes per 100 person-months, 95% CI: 0.1–0.7), with an incidence rate ratio (post- versus pre-) of 0.17 (95% CI: 0.07–0.40; *p* < 0.001). The odds of experiencing APE episodes were 74% lower (odds ratio: 0.26, 95% CI: 0.10–0.69) in patients from the post B-line implementation period compared with those from the pre-implementation period. Notably, adjusting for potential confounders did not affect the strength of this association, which remained statistically significant (*p* ≤ 0.030). Finally, dominance analysis indicated that the implementation of B-lines was the primary factor explaining the difference in APE episodes between the two periods, followed by dialysis duration and intra-dialysis weight gain. Conclusions: The systematic use of lung ultrasound (a simple, easy-to-learn, rapid and non-invasive method, easily performed at the patient’s bed) in everyday clinical practice was associated with a drastic reduction in episodes of APE in hemodialysis patients. Further observational and interventional studies are needed to confirm these results.

## 1. Introduction

In clinical practice, an accurate assessment of volume status represents a challenge. Volume overload is considered one of the most insidious risk factors for mortality and cardiovascular events in patients undergoing dialysis treatment, especially in patients with heart disease, a sub-category that represents approximately 30–40% of the entire dialysis population [1]. During the inter-dialytic period, fluid excess tends to accumulate in the lung area, causing progressive pulmonary congestion that impacts respiratory dynamics, the ventilation/perfusion ratio, and gas exchange [2,3]. Acute pulmonary edema (APE) is one of the most feared complications that generally requires an extra dialysis treatment in hemodialysis patients, but the extent of this problem is not well quantified. Only a few studies have reported on the incidence of this phenomenon in this population. Arneson et al. reported that in a dataset of 176,790 hemodialysis patients followed up for 2.5 years, 11% experienced fluid overload [4]. APE results in an increased rate of hospitalization. In fact, in another cohort of 215.251 admission hemodialysis patients included in the study by Plantiga et al., 10% were followed by an APE-related readmission within 30 days [5]. Although the pathophysiological mechanisms that lead to the accumulation of liquids in the interstitium are known, an accurate and easily applicable diagnostic method for its identification is lacking. In hemodialysis patients, traditional methods for identifying dry weight are not specific for pulmonary congestion or are not always applicable; examples include chest X-rays, which clearly involve exposure to ionizing radiation, as well as logistical and organizational difficulties; bioimpedancemetry, which identifies total body water but not thoracic fluid overload and which, in comparison with deuterium dilution (the gold standard for total body water), has a non-systematic error in 30–50% of cases, with an over- or underestimate of 3–5 L [6]; the evaluation of the inferior vena cava (VCI) and the collapsibility index to assess intravascular volume, which are operator-dependent, influenced by diastolic dysfunction, and do not reflect tissue and pulmonary overhydration [7]. Thermodilution provides information about the extravascular lung water (EVLW) [8,9]. The arterial thermodilution curve reflects the patient’s extravascular pulmonary water volume: the more the volume increases, the more the curve “stretches”. It is the golden standard for EVLW but, due to its invasive and complex characteristics, it cannot be used in clinical practice.

B-lines are ultrasound artifacts originating from water-thickened pulmonary interlobular septa. They are defined as a hyperechoic (laser-like) beam with a narrow base that originates from the pleural line and extends to the edge of the screen. They are formed by horizontal lines so small that they have a continuous aspect, and they move in sync with the respiratory movements of the lung and block the vision of other artifacts. The use of B-lines has been validated in the evaluation of pulmonary congestion in patients with cardiovascular disease [10] and in intensive care units, where it has high discriminatory power in identifying moderate and severe pulmonary congestion [11]. Agricola et al. describe the diagnostic accuracy of lung ultrasound in identifying alveolar imbibition and subclinical pulmonary edema compared with the thermodilution method [10]. Compared with clinical evaluation, a lung US-guided dry-weight adjustment protocol was associated with several strong outcomes in patients on chronic hemodialysis, such as a reduction in recurrent episodes of decompensated heart failure [12], a reduction in blood pressure levels and an improvement in echocardiographic parameters [13], and a reduction in intra-dialytic hypotensive episodes [14].

This single-center study aims to assess the incidence of APE episodes in a cohort of patients in whom the monitoring of volume expansion was supported by the use of B-lines in comparison with the incidence of the same events in another group of patients, whose management was entrusted to good clinical practice but without the aid of lung ultrasound. Another objective of the study was to rank the main risk factors impacting upon APE episodes by dominance analysis, a statistical technique used to assess the relative importance of a set of risk factors to explain the outcome variable [15].

## 2. Methods

The study protocol was approved by the ethical committee “Comitato Etico Regionale Sezione Area Sud—Reggio Calabria” (approval numbers 219 (24 February 2009) and 1357 (27 December 2012)).

### 2.1. Patients

All consecutive patients on chronic hemodialysis within the Nephrology Unit of the Grande Ospedale Metropolitano (GOM) of Reggio Calabria (Italy) were identified for inclusion and recruited. Two cohorts from 2 time periods were retrospectively considered. The first cohort (pre B-line implementation group; pre-BL-Group) was from the interval of 1 January 2007–31 December 2008, during which chest ultrasound had not yet been used, while the second cohort (post B-line implementation group; post-BL-Group) was from the time interval of 1 January 2017–31 December 2018, in which thoracic ultrasound had become frequently used as a diagnostic support in dialysis patients. For each cohort, patient and disease characteristics were reported. The 10-year interval between the two periods was specifically chosen to ensure that there was no overlap between the patients in the two cohorts, whereas the single-center design was adopted to minimize the influence of center effect on the study results.

### 2.2. APE Episodes

APE is defined as an episode characterized by sudden or worsening dyspnea associated with signs of salt and water overload, which is confirmed by chest auscultation or chest X-ray and which requires an additional dialysis session (reference). Events due to infectious or irritating problems were excluded. By accurately reviewing the electronic medical records, all episodes of APE were recorded To evaluate the incidence of APE in the two cohorts, for each patient, the period at risk (months of exposure to dialysis in the interval considered) was calculated.

### 2.3. Chest Ultrasound and Dry-Weight Prescription

**Figure 1:**
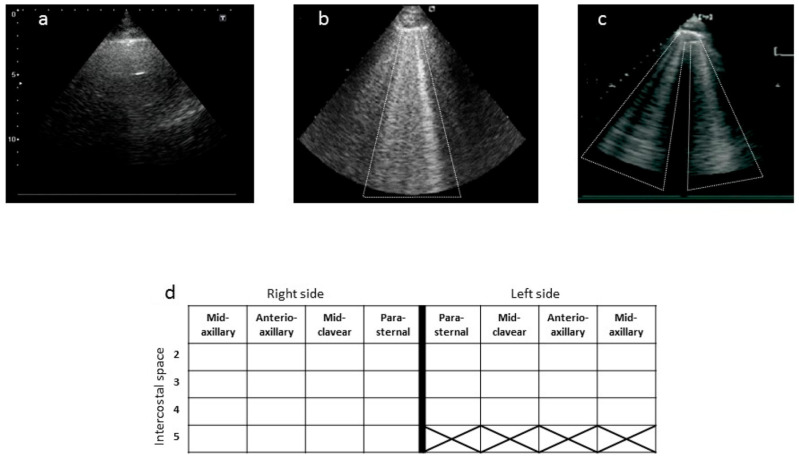
In the figure, three chest ultrasound images are shown. (**a**) Absence of B-lines. In (**b**,**c**), moderate and severe pulmonary congestion, respectively, are reported, (**d**) case report form for US B-lines data collection.

Chest ultrasound was performed using the anterior-to-lateral approach according to Gargani L. 2011 [16]. For each space, a score ranging from zero (absence of B-lines) to ten B-lines (maximum assignable score) can be assigned [16] (see Figure 1). In our Nephrology Unit, the dry weight was established on an individual basis by considering traditional criteria such as the presence/absence of peripheral edema and/or crackles at the lung bases, blood pressure values, patient’s weight, and symptoms (cramps and hypotension). Lung water assessment was performed by a chest US performed by the attending nephrologist before and/or after a hemodialysis session, and the lung scan was used to titrate the ultrafiltration [12]. In patients with moderate to severe lung congestion (>15 US-B-lines), ultrafiltration was intensified, and this was followed by periodical lung US until the treatment goal (<15 US-B-lines) was achieved. The B-line assessment was performed using any ultrasound machine available at the time, provided that it was equipped with a cardiac probe with a frequency generally between 2.5–3.5 MHz. A YouTube video showing how to measure US B-lines is available at (https://www.youtube.com/watch?v=amsULLws8GI (accessed on 17 April 2009)).

### 2.4. Statistical Analysis

Data were summarized as the mean ± standard deviation, median and interquartile range, or absolute number and percentage, as appropriate. Between the groups, differences were assessed by the *t*-test, the Mann–Whitney U test or the chi-squared test, depending on the nature and distribution of the variables. We also used the standardized mean difference (SMD) together with the *p*-value to assess between-group differences in baseline characteristics. When the sample size is relatively small, such an approach helps to identify more variables as potential confounders than those identified by a *p*-value ≤ 0.05. This is because meaningful differences can be quantified as having no statistical significance despite their clinical relevance. The SMD provides a measure of effect size independently of sample size, allowing for a more meaningful interpretation of the magnitude of baseline differences between groups. In a pairwise comparison, an SMD > 0.10 was considered clinically relevant. The association between pre- versus post B-lines implementation and the frequency of APE was assessed by crude and adjusted logistic regression models, including all the variables with a between-group SMD > 0.10. To avoid collinearity between pre- and post-dialysis BPs, two multivariable logistic regression models were fitted: one including pre-dialysis BPs and one including post-dialysis BPs. In these models, data were expressed as an odds ratio, 95% confidence interval (CI), and *p*-value. The relative contribution of B-line implementation on the occurrence of APE episodes was also assessed by dominance analysis, i.e., a statistical technique used to assess the relative importance of risk factors on a specific outcome variable in a multivariable regression model [15]. In the context of logistic regression, dominance analysis helps to determine the contribution of each independent variable to the overall model fit by comparing the incremental value added by each variable across all possible subset models. To perform dominance analysis, we first fit a series of logistic regression models, each including different combinations of the predictor variables. For each model, a measure of the model fit is calculated, such as the pseudo R-squared value. The incremental contribution of each variable is then assessed by examining the change in the model fit statistic when the variable is added to models that do not include it. The incidence rate of APE was expressed as episodes per 100 patient-years (and 95% CI) and the between-period difference was expressed as the incidence rate ratio and 95% CI.

Furthermore, to assess the potential impact of unmeasured confounders on the effect of B-line implementation between the two periods on the risk of APE (≥4 episodes per 100 person-months), we conducted a scenario analysis. This analysis considers the effects of unmeasured confounders of varying magnitudes (ranging from 10% to 60%) on the protective effect of B-line implementation on the risk of APE. Statistical data analysis was performed by STATA for Windows (Version 16), Texas, USA.

## 3. Results

The overall study population was composed of 206 hemodialysis patients. Among these, the pre- and post-B-line implementation groups were composed of 98 patients (85 prevalent and 13 incident patients followed up between 1 January 2007 and 31 December 2008) and 108 patients (81 prevalent and 27 incident patients followed up between 1 January 2017 and 31 December 2018), respectively. Patients of the post-B-line implementation group were older; had a higher New York Heart Association Functional Classification (NYHA class); more frequently presented with Cardiovascular (CV) comorbidities in their clinical history and a higher Kt/V and post-dialysis systolic BP; and displayed a longer dialysis duration and lower intra-dialysis weight gain, pre-dialysis systolic BP, and pre- and post-diastolic BPs compared with those from the pre-B-line implementation group (Table 1) (all |SMD| > 0.10). Most patients from the post-B-line implementation group were treated with bicarbonate dialysis (84%) while only a minority of these patients were treated with hemodiafiltration (12%) or other dialysis technique (4%) (all |SMD| > 0.28). No difference was observed between the two groups for the follow-up duration (Table 1).

### 3.1. Episodes of APE

Overall, 44 episodes of APE occurred in 24 patients from the two cohorts. APE episodes occurred more frequently in patients of the pre- (18/98, i.e., 18.4%) than in those of the post- (6/108, i.e., 5.5%) B-line implementation group (*p* = 0.004). An analysis based on repeated events of APE (see Table 2) showed that the incidence rate of APE was much higher during the pre-B-line implementation period (2.0 APE episodes per 100 person-month, 95% CI: 1.4–2.7) than during the post-B-line implementation period (0.3 APE episodes per 100 person-month, 95% CI: 0.1–0.7), with an incidence rate ratio (post- versus pre-) of 0.17 (95% CI: 0.07–0.40; *p* < 0.001)].

### 3.2. Logistic Regression and Dominance Analyses

**Figure 2:**
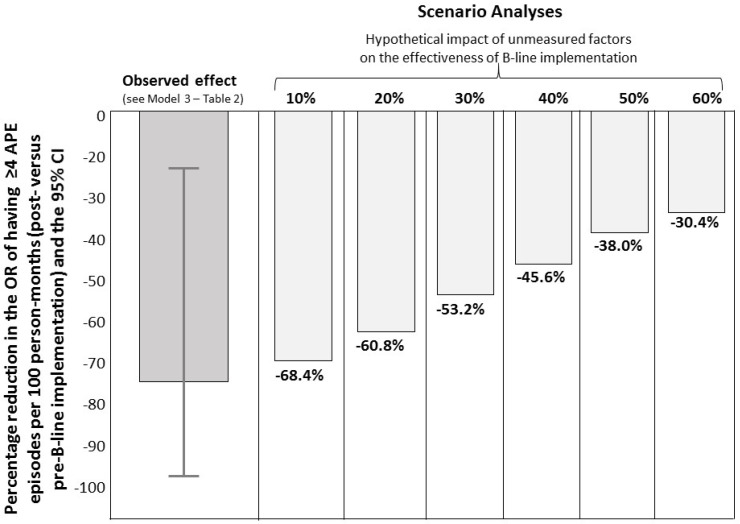
Scenario analyses of the potential impact of unmeasured factors on the effect of B-line implementation and the risk of EPA (see the Section 3 for more details).

The odds ratio of having APE episodes was 74% lower (odds ratio: 0.26, 95% CI: 0.10–0.69) in patients of the second (post-B-line implementation period) than in those of the first group (pre-B-line implementation period) (Table 3; Crude Model). Of note, data adjustment for the measured confounders (i.e., for variables that differed (|SMD| > 0.10) between the two groups; see Table 1) did not affect the strength of this association (see Table 3; Models 1–2), which remained statistically significant (*p* ≤ 0.030). Of note, the inclusion of the NYHA class in Table 3 (Models 1–2) further amplified the effect of B-line implementation on APE episodes (odds ratio: 0.14, 95% CI: 0.04–0.51; *p* = 0.003 and odds ratio: 0.16, 95% CI: 0.05–0.58; *p* = 0.005). A scenario analysis considering the effects of unmeasured confounders of varying magnitudes (from 10% to 60%) on the benefit of B-lines in reducing the risk of EPA showed that the protective effect of B-line implementation ranged from 68.4% (the best scenario) to 30.4% (the worst scenario) (Figure 2). To assess the relative importance of B-line implementation on the occurrence of APE episodes in the two study periods, we performed two dominance analyses: one based on pre-dialysis and one based on post-dialysis BPs (Table 4A,B). These analyses consistently indicated that B-line implementation was the first factor in rank order that explained the difference in APE episodes between the two periods, followed by dialysis duration and intra-dialysis weight gain (Table 4A,B).

## 4. Discussion

This single-center study suggests that the implementation of ultrasound B-lines was associated with a significant reduction in the incidence of APE episodes in a single-center study, underscoring the importance of chest ultrasound for improving patient outcomes in the dialysis setting.

In patients on dialysis, APE episodes are often the expression of cardiac injury as well as of volume expansion, and it is associated with cardiovascular morbidity and mortality. The management of APE episodes is complex, requiring additional dialysis sessions with the simultaneous presence of both a nephrologist and a nurse. It also involves additional risks associated with the use of vascular access (arteriovenous fistula or central venous catheter) and leads to increased healthcare expenditure. An analysis conducted in the United States demonstrates an additional cost per single episode of APE of more than USD 6000 [4]. APE is rarely chosen as an outcome, being more frequently replaced by outcomes considered more robust, such as myocardial infarction or death, despite its clinical, organizational, managerial, and cost impact. The results of this study show that independently of major potential confounders (such as age, background CV comorbidities, intra-dialysis weight gain, pre- and post-dialysis BPs, dialysis duration, Kt/V, and treatment modality), the number of episodes of APE was significantly reduced following the implementation of the use of thoracic ultrasound. Of note, such an effect has a magnitude similar to that observed in a secondary analysis of a randomized clinical trial testing the effect of B-lines versus the standard of care in recurrent episodes of decompensated heart failure [12]. Furthermore, the dominance analysis conducted to assess the relative importance of B-line implementation on the occurrence of APE episodes revealed that B-line implementation was the most significant factor explaining the reduction in APE episodes, followed by dialysis duration and intra-dialysis weight gain.

Thoracic ultrasound is a simple, easy-to-learn, non-invasive, and rapid method that is well tolerated by patients, as it lasts an average of 2 min, with an intra- and interobserver variability of 5% and 7%, respectively [17]. It is worth noting that it has the power to provide early identification of a pulmonary edema: in the case of water overload, the accumulation of liquids will start at the interstitial level, causing a thickening of the subpleural interlobular septa, which hinders the propagation of the ultrasound beam, thus generating a difference in acoustic impedance between the liquid and air, which allows the formation of the ultrasound image of the pulmonary comet. In clinical terms, this provides a great advantage for intervention because, keeping in mind that interstitial pulmonary edema precedes alveolar pulmonary edema, the finding of pulmonary comets is a very early marker of pulmonary edema. The results of this study demonstrate that the support provided by B-lines in the assessment of volume expansion is informative for maintaining better dry-weight management of the hemodialysis population, resulting in a reduction in emergency dialysis and a containment of healthcare costs. Management of the hydration status requires continuous attention from healthcare personnel. As demonstrated in other processes requiring continuous commitment, such as the use of gloves during the management of vascular access in pediatric patients, this clearly increased in the period of active surveillance while decreasing over time when attention was reduced [18].

### Strengths and Limitations of the Study

Our study has points of strength. The study design includes a 10-year interval (2007–2008 and 2017–2018) between the pre- and post B-line implementation periods, ensuring no overlap between the two patient cohorts. On the other hand, within the same decade, there were no major significant changes in the clinical policy for preventing episodes of acute pulmonary edema beyond the application of ultrasound B-lines. By conducting the study within a single center, we avoided the influence of center-specific practices and variations in care. This design choice enhances the internal validity of the study, making the results more reliable and attributable to the implementation of lung ultrasound rather than to differences in clinical practices across multiple centers. The study employs comprehensive statistical methods, such as multivariable logistic regression analysis, adjusting for major confounders and dominance analysis. It focuses on a well-defined and clinically significant outcome—the incidence of APE episodes—which were accurately collected by reviewing electronic medical records. The implementation of lung ultrasound as a routine practice is a practical, easy-to-learn, rapid, and non-invasive method that can be easily performed at the patient’s bedside.

Despite these strengths, this study also has limitations that should be acknowledged. The observational nature of the study inherently limits the ability to establish causality. While we observed a significant reduction in APE episodes following the implementation of B-lines, we cannot exclude the possibility that other unmeasured factors may have contributed to the observed outcomes. The lack of randomization in assigning patients to the pre- and post-implementation groups limits the ability to control for confounding variables. Although we adjusted for potential confounders in our analysis, residual confounding cannot be entirely ruled out. In this regard, it is important to note that a decade in medicine can bring about significant advancements and changes in clinical practice, which could introduce unmeasured confounding factors. Advancements such as blood volume tracking systems (BVM; HemoControl) and other biofeedback programs, increasingly implemented over the past decade, could influence cardiovascular outcomes and the hemodialysis performance. These factors were not directly measured in our study, representing an intrinsic limitation. To address this concern, we conducted a scenario analysis to evaluate the potential impact of unmeasured factors on the effectiveness of B-line implementation in reducing the risk of acute pulmonary edema. By adopting these scenarios, we found that the protective effect of B-line implementation ranges from 68.4% (in the best-case scenario) to 30.4% (in the worst-case scenario). This suggests that while other advancements in medical technology and practice may contribute to improved outcomes, the implementation of B-lines remains a significant factor in reducing the risk of acute pulmonary edema. Finally, the single-center nature of the study can jeopardize the generalizability of the study results.

## 5. Conclusions

In conclusion, while the study provides valuable insights into the potential benefits of B-line implementation in reducing APE episodes among dialysis patients, the limitations inherent to its observational design and temporal differences between the study groups should be considered when interpreting the findings. Future randomized controlled trials are needed to confirm these results and to establish a causal relationship between B-line implementation and improved clinical outcomes in this patient population.

## Figures and Tables

**Table 1:** Main demographic and clinical data by patient groups.

	B-Line Implementation			
	Pre	Post		
	Group 1 (*n* = 98)	Group 2 (*n* = 108)	*p*-Value	|SMD|
Age (years)	64 ± 15	67 ± 14	0.06	0.26
Duration of renal dialysis treatment (years)	3.8 (1.7–7.6)	3.2 (1.3–7.8)	0.84	0.03
Male sex, *n*. (%)	64 (65%)	67 (62%)	0.62	0.07
Diabetes, *n*. (%)	21 (21%)	27 (25%)	0.54	0.08
Background CV comorbidities, *n*. (%)	19 (19%)	33 (31%)	0.07	**0.26**
Smokers, *n*. (%)	28 (29%)	30 (28%)	0.90	0.02
Hypertension, *n*. (%)	73 (74%)	77 (71%)	0.61	0.07
New York Heart Association classification (NYHA class)	1 (1–2)	2 (1–3)	<0.001	**0.62**
Pre-dialysis body weight (kg)	67 ± 17	67 ± 15	0.92	0.01
Post-dialysis body weight (kg)	65 ± 17	65 ± 15	0.82	0.03
Intra-dialysis weight gain (kg)	2.3 ± 0.7	2.1 ± 0.6	0.003	**0.41**
Pre-dialysis systolic BP (mmHg)	138 ± 15	135 ± 17	0.24	**0.16**
Pre-dialysis diastolic BP (mmHg)	73 ± 9	70 ± 10	0.02	**0.32**
Post-dialysis systolic BP (mmHg)	128 ± 16	133 ± 19	0.047	**0.28**
Post-dialysis diastolic BP (mmHg)	71 ± 9	70 ± 9	0.33	**0.14**
Dialysis duration (minutes)	225 ± 15	232 ± 11	<0.001	**0.58**
Kt/V	1.30 ± 0.25	1.40 ± 0.29	0.007	**0.38**
Bicarbonate dialysis, *n*. (%)	98 (100%)	91 (84%)	<0.001	**0.60**
Hemodiafiltration, *n*. (%)	0 (0%)	13 (12%)	<0.001	**0.52**
Other treatment modalities, *n*. (%)	0 (0%)	4 (4%)	0.05	**0.28**
Follow-up duration (months)	24.1 (12.0–24.1)	24.1 (12.1–24.1)	0.78	**0.04**

|SMD|: standardized mean difference as absolute values. The bold data indicate between-group differences with an |SMD| > 0.10.

**Table 2:** Episodes of APE.

	B-line Implementation	
	Pre	Post
	Group 1 (*n* = 98)	Group 2 (*n* = 108)
Patients with APE (number)	18	6
Patients with 1 episode of APE	5	5
Patients with 2 episodes of APE	9	1
Patients with 3 episodes of APE	2	0
Patients with 4 episodes of APE	2	0
Episodes of APE (number)	37	7

**Table 3:** Logistic regression analyses. Dependent variable: patients ≥ 4 APE episodes per 100 person-months (the threshold corresponding to the lowest incidence rate value in patients experiencing such an event).

	Crude Model	Model 1	Model 2
	Odds Ratio (95% CI); *p*-Value	Odds Ratio (95% CI); *p*-Value	Odds Ratio (95% CI); *p*-Value
Post- Versus Pre-B-Line Implementation	0.26 (0.10–0.69); *p* = 0.007	0.27 (0.08–0.88); *p* = 0.030	0.24 (0.07–0.78); *p* = 0.017
Age (years)		1.03 (0.98–1.09); *p* = 0.18	1.03 (0.99–1.08); *p* = 0.17
Background CV comorbidities (yes versus no)		2.66 (0.96–7.36); *p* = 0.06	2.73 (1.04–7.19); *p* = 0.042
Intra-dialysis weight gain (kg)		2.43 (1.31–4.51); *p* = 0.005	2.50 (1.35–4.62); *p* = 0.003
Pre-dialysis systolic BP (mmHg)		1.03 (0.99–1.07); *p* = 0.18	
Pre-dialysis diastolic BP (mmHg)		0.97 (0.92–1.04); *p* = 0.42	
Post-dialysis systolic BP (mmHg)			1.02 (0.99–1.06); *p* = 0.23
Post-dialysis diastolic BP (mmHg)			0.98 (0.91–1.05); *p* = 0.55
Dialysis duration (minutes)		0.96 (0.92–0.99); *p* = 0.010	0.96 (0.93–0.99); *p* = 0.016
Kt/V		2.04 (0.25–17.01); *p* = 0.51	1.95 (0.27–14.35); *p* = 0.51
Treatment modality (bicarbonate versus HDF/other)		0.98 (0.10–9.83); *p* = 0.99	0.84 (0.09–7.66); *p* = 0.87

Model 1: includes pre-dialysis systolic and diastolic BPs; Model 2: includes post-dialysis systolic and diastolic BPs.

**Table 4:** Dominance analysis, including pre-dialysis systolic and diastolic BPs (**A**) and post-dialysis systolic and diastolic BPs (**B**). (**A**) Dependent variable: patients with ≥ 1 APE episode per 100 person-months. (**B**) Dependent variable: patients with ≥ 1 APE episode per 100 person-months.

**(A)**		
	**Dominance Analysis Based on Pre-Dialysis Systolic and Diastolic BPs**	
	Standardized Dominance Statistics (%)	Ranking
B-Line implementation	29.1%	1
Dialysis duration	20.8%	2
Intra-dialysis weight gain	14.0%	3
Pre-dialysis systolic BP	11.0%	4
Background CV comorbidities	10.2%	5
Age	10.0%	6
Pre-dialysis diastolic BP	2.0%	7
Kt/V	1.5%	8
Treatment modality	1.4%	9
**(B)**		
	**Dominance Analysis Based on Post-Dialysis Systolic and Diastolic BPs**	
	Standardized Dominance Statistics (%)	Ranking
B-Line implementation	32.3%	1
Dialysis duration	21.1%	2
Intra-dialysis weight gain	15.9%	3
Background CV comorbidities	10.9%	4
Age	10.5%	5
Post-dialysis systolic BP	5.2%	6
Kt/V	1.6%	7
Post-dialysis diastolic BP	1.3%	8
Treatment modality	1.2%	9

## Data Availability

The datasets presented in this article are not readily available because they are protected by privacy. Requests to access the datasets should be directed to vincenzoantonio.panuccio@ospedalerc.it.
